# Acquired Resistance to Tuberculosis: A Factor in Clinical Type and Prognosis
1Address delivered to the Bristol Medico-Chirurgical Society, Dec. 13th, 1922.


**Published:** 1923-01

**Authors:** S. Lyle Cummins

**Affiliations:** David Davies Professor of Tuberculosis, University of Wales; Principal Medical Officer, Welsh National Memorial Association; lately Professor of Pathology, R.A.M. College, London


### ACQUIRED RESISTANCE TO TUBERCULOSIS: A FACTOR IN CLINICAL TYPE AND PROGNOSIS.[1]

BY

S. Lyle Cummins, C.B., C.M.G., M.D.,

*David Davies Professor of Tuberculosis, University of Wales; Principal Medical Officer, Welsh National Memorial Association; lately Professor of Pathology, R.A.M. College, London.*

---

It is appropriate, here in Bristol, the city of William Budd, to address a medical audience on the subject of tuberculosis, a disease for ever associated with his name.

If the responsibilities of a large practice and the study of typhoid fever and other diseases closed for William Budd the paths of experimental investigation and research that were so successfully followed by his great French contemporary, Villemin, these preoccupations were powerless to confine the independent play of thought, the informed and

[1] *Address delivered to the Bristol Medico-Chirurgical Society, Dec. 13th, 1922.*

original speculation that led this great Englishman to the same conclusions which Villemin by experiment demonstrated to be true. He writes as follows in 1867 :—

"The idea that phthisis is a self-propagated zymotic disease, and that all the leading phenomena of its distribution may be explained by supposing that it is disseminated through society by specific germs contained in the tuberculous matter cast off by persons already suffering from the disease, first came into my mind, unbidden, so to speak, while I was walking on the Observatory Hill at Clifton, in the second week of August, 1856."

Tuberculosis an infective disease ; there was the truth that had flashed into the mind of Budd. It may seem a truism to-day, but we must remember that Budd spoke before Villemin had proved that tuberculous material could reproduce the disease in animals and before Robert Koch had isolated the tubercle bacillus. Against the theory of infection was the constantly observed fact that persons might be closely associated with a tuberculous patient for long years without ever developing the disease. At that time there was no Von Pirquet reaction to show that infection may exist without illness ; there were no records such as those of Naegeli, of Bughart, or of Hamburger to prove that a large percentage of cadavers show evidence of old and obliterated tuberculous infection.

When we take into account the state of knowledge on this subject in 1856, we are able to realise the wonderful vision that led William Budd with unerring perception straight to the truth.

What was the line of thought that led him ? It was the consideration of the phenomena of the disease when introduced into isolated communities such as the population of certain islands in the Pacific, previously free from all contact with infection. Budd realised that only under these circumstances could the disease manifest clearly its infectious characters, because here the people were "virgin soil" to the infective agent. He realised, then, though perhaps without expressing it even to himself, that contact with infection may lead to resistance, and that in the civilised and urbanised communities of this country the spread of infection is masked by another and an allied factor, the acquisition of resistance.

If we glance into the wards of an isolation hospital where cases of typhoid fever are being treated we can say with confidence that of those present, now lying too ill to read or talk, all will have left the institution in a month or two; from 15 to 20 per cent. may perhaps be dead, the rest clinically well, but with one or two in every hundred doomed to become "typhoid carriers" for the rest of their natural lives.

But if we look, instead, into a ward full of advanced cases of pulmonary tuberculosis we are led to make a very different forecast. In a month or so nearly all those present to-day will still be present, still propped up in bed knitting or reading the papers, still hopeful that their health will soon be restored. It will be a year before 15 or 20 per cent. are dead, but nearly all of them will be dead five years hence. And those who recover sufficiently to leave the hospital during the interval will all be "tubercle carriers," excreting the germs, not only in urine or fæces where its safe disposal is the rule, but also into the circumambient air that must be breathed by their fellow-creatures.

This contrast, though obvious, is important. It marks out tuberculosis as a disease with few parallels in human pathology, as a disease infection which must be almost universal under conditions of close human contact, as something to which civilised man must adapt himself or perish.

In the case of most of the bacterial diseases of man the issue is not long in doubt. The bacterial invasion is either arrested and overcome, with recovery of the patient, or else prevails so completely as to lead to a fatal result. There is little or no tendency to a delicate and protracted state of balance between the invasive powers of the germ and the defensive powers of the body.

For those pathological conditions in which such a state of " balance " is common I desire to use the expression " competitive symbiosis " to describe the relations of the parasite to the host.

There are certain protozoal and spirochætal parasites which have the power of settling down in " competitive symbiosis " with their vertebrate hosts. Amongst bacterial parasites, however, it is only the tubercle bacillus and the closely allied germs of leprosy, glanders, actinomycosis and a few other streptothrices and fungi that possess this formidable character. They owe it to their relatively low virulence on the one hand and to their powers of resistance to the self-sterilising properties of the human body on the other.

It is only germs of relatively low virulence combined with considerable powers of survival in the tissues that can settle down in " competitive symbiosis " in the human or animal body, and in the case of such germs the character of the " symbiosis " will vary with variations in the mutual adaptability of parasite and host.

Taking, for instance, the human tubercle bacillus, the " competitive symbiosis " is one in which the " competitive efficiency " of the parasite varies with the species of animal host in which it finds itself. In the cow or the rabbit the " competitive efficiency " of the host outstrips that of the parasite, the latter being gradually eliminated. In the monkey or guinea-pig the parasite wins and the animal host perishes. In man the parasite and the host are so evenly matched that accidental factors, such as amount of the initial or subsequent doses of infective material, the age of the host, or an environment favourable or the reverse, may finally decide the issue.

But these accidental factors cannot be other than indirect in their action. They bring about their effects through their influence on a much more intricate machinery. In the final resort it is the competitive powers of the host and the parasite respectively that matter. A man may be as susceptible as the guinea-pig or as resistant as the ox to the human tubercle bacillus ; and it is my thesis that this state of susceptibility or resistance depends not on inborn characters, as in the case of the guinea-pig or ox, but upon the want of, or development of, a relative immunity, only to be acquired through successful competition with the germ.

That such a relative immunity can be acquired even by susceptible animals has been proved by Calmette and others working with bacilli of reduced virulence. All that is necessary is that the " competitive efficiency " of the parasite shall be reduced below that of the host by prolonged culture on bile media, and relative immunity instead of fatal disease follows inoculation of the living and attenuated culture.

At this point it may, perhaps, be suggested that these considerations have their bearing in preventive rather than in clinical medicine. A moment's thought will show that this is not the case. Much work is still necessary before we shall be justified in advocating " living vaccines " in the prevention of tuberculosis, and it is only with living bacteria that satisfactory prophylactic immunity has as yet been attained. But with regard to the symptomatology of the disease, the importance of these concepts appears to me to be fundamental.

The reason of this is that bound up with and forming part of the processes of acquired immunity is the character of "reactivity," "hypersensitivity" or "allergy," that character which makes the tissues of the host intolerant towards the bacterial protoplasm of the parasite, and which therefore determines the symptoms of the disease.

Let us briefly consider the applicability of this concept to tuberculosis.

The first point that requires to be emphasised is that the so-called "toxin" of tubercle bacilli, tuberculin, is without appreciable effect on the uninfected human or animal body. Relatively large quantities can be inoculated into the newly-born infant or into the guinea-pig without causing any symptoms at all.

It is only when the human or animal body has been rendered intolerant by infection with the tubercle bacillus that tuberculin becomes for it a toxic substance. In other words, tuberculin causes no symptoms by itself. It only leads to symptoms when the cells or humours of the body react to it.

It was the discovery of this fact through the severe local reaction noted on inoculating dead tubercle bacilli into an already infected guinea-pig (Koch's phenomenon) that led Robert Koch to search for and ultimately to produce tuberculin.

As with tuberculin, so with tubercle bacilli, no symptoms are produced except through the reaction of the host.

If a young guinea-pig is inoculated with a large dose of living human tubercle bacilli there is no immediate symptom of illness. The weight continues to increase, the animal feeds as usual, and for some days there is nothing to suggest that the guinea-pig is doomed. But by degrees the tissues begin to react, the temperature becomes irregular and the weight begins to fall. At or shortly before this moment an intra-dermal inoculation of tuberculin will evoke a local reaction. The animal has become "intolerant" or "allergic," and this

"intolerance" appears to determine the symptoms of infection.

In an already infected guinea-pig the effect of a further inoculation is not slow in appearing, but is rapidly manifested by a massive local swelling with a tendency to caseation and breaking down so as to form a fistula.

Reaction, then, takes the form of rapidly produced local inflammation at the point where the antigen has been injected, together with certain symptoms that depend upon the products of the interaction between the parasite and the cells or humours of the host.

This inflammatory reaction tends to "localise" the germs and keep them immured at the point of reaction by the building around them of the cellular barriers seen in every "tubercle."

Giant cells have been reported by Wolff Eisner as present at the site of a Von Pirquet skin reaction, thus suggesting the close relation between the tuberculin and the bacteria that produce it as well as between the reaction to each.

Here we arrive at a conception of great importance. Where there is resistance the allergic reaction eventuates in changes which tend to localise the germs and keep them "localised," symptoms, both local and general, being definite. Where there is no resistance, no reaction, the germs are able to disseminate widely from the point of entry, the symptoms at first being slight or absent.

The application of these conceptions to the interpretation of symptomatology in human tuberculosis is full of interest.

It is common knowledge that, apart from a small number of exceptional cases, the general symptomatology of tuberculosis shows well-marked differences in different age-groups in this country.

In early infancy the disease is seldom recognised, so insidious is its clinical course and so few its symptoms; and yet it is nearly always generalised and tends to be fatal.

In childhood the presence of extensive infection may fail to give rise to any definite symptoms until some local reactive process occurs. The characteristic feature of tuberculosis in childhood, apart from the frequency with which it affects lymphatic glands, is its tendency to become generalised through the body. As a result we find in childhood that, apart from adenitis the commonest lesions are tuberculous meningitis and affections of the bones and joints, lesions that can only occur after the generalisation of the infection, chiefly by way of the blood-stream.

In these generalised infections in childhood the constitutional disturbance is frequently minimal. A glance into the wards at Alton, Oswestry or Carshalton will reveal dozens of well-nourished, rosy children immobilised on Thomas's splints or other appliances because of extensive tuberculosis of the spine, the hips, the knee, or perhaps all three at once—generalised tuberculosis with hardly any symptoms. Like the inoculated guinea-pigs, their weight is steadily rising although they are infected with a general disease. Unlike the guinea-pigs, they will probably continue to increase in weight, and with appropriate orthopædic treatment finally completely recover, probably because the initial dose was, in them, not relatively so massive as that injected into the guinea-pig.

In other words, the tuberculosis of infancy and childhood is in general a disease of non-allergic tissues, a disease that gives rise to few constitutional symptoms and yet tends to diffuse widely throughout the body.

The tuberculosis of adults, on the other hand, exhibits the character of "intolerance." Localisation in the lungs, with inflammatory phenomena around the foci, is the rule; and with this tendency to localisation goes a symptomatology which, though liable to variations, is still so definite that the constitutional symptoms are often of themselves more valuable in diagnosis than physical signs. This is the tuberculosis of allergic tissues.

Between childhood and adult age lies the period of adolescence, and it is here that the two main factors of infection and resistance are subject to violent disadjustments of balance. It is at this stage of rapid growth and severe physiological strain that the infections contracted during childhood and still existent in lymphatic nodes and other foci tend to assume fresh activity; and it is at this period that the individual, quitting the shelter and isolation of home, is exposed to the risk of close contact with infective persons in the course of business or education. The resistance at this stage appears to be, in many cases, just sufficient to localise the infection in the lungs and to evoke symptoms, but insufficient to prevent the rapid spread of the disease locally by the extension and coalescence of foci. Thus it comes about that, especially in the case of young females just emerged from the relative isolation of home life, and of young lads from country districts, we frequently encounter a very rapid and fatal type of pulmonary tuberculosis—the clinical entity that underlies the "young adult" type of mortality to which the statistical researches of John Brownlee have directed attention.

The pathological changes that express these different phases of acquired resistance or the absence of it are very characteristic.

It seems certain that in the virgin soil of infantile tissues the tubercle bacillus can pass through mucous membranes and along lymphatic channels without exciting any reaction at all in the first instance. It is only when arrested in the nearest lymph node that the germ begins to multiply and to poison surrounding cells by its products, with the result that its antigenic effects become operative in generating some degree of resistance.

This process can be followed in the guinea-pig by the installation of a drop of living bacillary emulsion into the conjunctival sac. There is at first no conjunctivitis, but the animal develops cervical adenitis and then general tuberculosis. In an experiment of this nature recently carried out by me the eye became acutely inflamed towards the end of the fourth week, a few residual bacteria perhaps exciting an allergic reaction when a measure of resistance had developed.

In tissues that have become slightly resistant, but not sufficiently so to control and limit the spread of infection in the ineffectively allergic soil of the inoculated guinea-pig for instance, the presence of tubercle bacilli tends to excite the formation of extensive areas of homogeneous granulation tissue not unlike those characteristic of the lesions in actinomycosis. This stage is seldom seen at necropsy in human subjects, as the infection has usually lasted long enough, even in infants, to excite the formation of characteristic tubercles surrounded by a zone of cellular reaction, or to the further stage of fusion of foci, death of tissues and caseous degeneration of fairly extensive areas.

In the highly resistant individual the cellular proliferation soon leads on to the formation of fibrous tissue, and to the isolation of infected areas within cellular and fibrous barriers that are often very successful in limiting the spread of the disease, though they may themselves cause mechanical damage and inconvenience, and may even assist secondary infections by shutting out the blood and humours from diseased foci.

The conception, then, that I wish to express is that behind clinical type in tuberculosis lie these two main factors of infection and acquired resistance, upon the relative preponderance of which largely depend the manifestations of the disease.

To revert to my conception of "competitive symbiosis," where the "competitive efficiency" of the parasite is greatly in excess the progress of the disease will be rapid and the tendency to generalisation of infection will be well marked: where, on the other hand, the "competitive efficiency" of the host is excessive the infection will be either eliminated before it can even make its presence known or else localised by fibrous barriers and unable to progress. In between these extremes will be found the majority of clinically recognisable cases, and in prescribing treatment or in attempting to forecast the issue of the disease the wise physician will give close attention to the degree of resistance possessed by the patient. There is a common tendency to lay too much stress on physical signs and too little on more subtle indications. The real clue to the case lies in the patient's responses to auto-inoculation on the one hand and to rest on the other, these responses being considered in relation to the extent of the disease. In my opinion Inman's classification is one which should be applied mentally by every physician to every case of pulmonary tuberculosis with which he has to deal. If a patient is "resting febrile" it may be safely assumed that the competitive efficiency of the parasite is in excess, and the prognosis must be very guarded even if the physical signs are minimal. The "ambulant afebrile" case, even with extensive disease, is one in which a high degree of resistance has been acquired. That it has been acquired is certain, for had it existed as an inborn character the degree of resistance that can even now curb and control extensive disease would have been sufficient to prevent its development in its early stages.

For the "ambulant febrile" patient the issue is in the balance. It is here that the judgment of a skilled physician finds its highest opportunity.

Finally, let it be remembered that a high degree of resistance may be temporarily masked by a still higher degree of infection.

No case is quite hopeless in this disease, though hope may seem ever so slight. We all meet now and again patients such as Cases xxiv. and xxv. of Lænnec's series in whom desperate symptoms gave place to restored health, though we may be permitted to find the explanation in acquired resistance rather than in "sulphur baths, astringents and asses' milk."

